# The Antifungal Effect of Propolis Endodontic Irrigant with Three Other Irrigation Solutions in Presence and Absence of Smear Layer: An *In Vitro* Study

**DOI:** 10.22037/iej.v13i2.19227

**Published:** 2018

**Authors:** Lama Awawdeh, Ahmed Jamleh, Maha Al Beitawi

**Affiliations:** a *Department of Conservative Dentistry, Faculty of Dentistry, Jordan University of Science and Technology, Irbid, Jordan*

**Keywords:** Candida albicans, Chlorhexidine, Irrigating Solution, Propolis, Smear Layer, Sodium Hypochlorite

## Abstract

**Introduction:**

The aim of this *in vitro* study was to compare the antifungal effect of propolis as an endodontic irrigant agent with a mixture of doxycycline, citric acid, and a detergent mixture (MTAD), 2% chlorhexidine (CHX) and 3% sodium hypochlorite (NaOCl) against *Candida albicans *in presence and absence of smear layer.

**Methods and Materials:**

Extracted teeth with single canals (*n*=104) were prepared and randomly distributed into four experimental groups; 30% propolis, MTAD, 2% CHX and 3% NaOCl. Each group had two subgroups; with and without smear layer. The antifungal effectiveness was evaluated. The Kruskal-Wallis and Mann-Whitney tests were used to compare the overall effectiveness of different treatments at significance level of 0.05.

**Results:**

Propolis, CHX and NaOCl had similar levels of effectiveness to each other against *C. albicans*, and these levels were not affected by the presence or absence of the smear layer. Each irrigant was significantly more effective than MTAD or saline solution. MTAD was less effective in the presence of the smear layer than in its absence.

**Conclusions:**

Propolis irrigation can produce root canals that are free of *C. albicans*, even in the presence of the smear layer.

## Introduction

Endodontic treatment is performed to eradicate infections that have spread throughout the root canal system as result of microbial invasion. It involves the shaping and cleaning of the root canal system using endodontic instruments, irrigants and medicaments. The use of effective antimicrobial irrigants is essential to effectively eliminate bacteria and fungi from the infected root canals, and so to achieve treatment success [1].

*Candida albicans *is one of the most common species in the oral cavity, in both healthy and medically compromised individuals [1]. Its prevalence has been shown to range from 7% to 55% in infected root canals [2]. Generally, *C. albicans *is present as a persistent species, most often in filled root canals, where it can survive even in harsh ecological conditions [1, 3]. It can be isolated from the root canal either in pure culture [4] or together with bacteria [5, 6]. *C. albicans *is considered to be a dentinophilic micro-organism with the ability to invade dentinal tubules [7] and to use dentin and the smear layer as sources of nutrition [8]. Smear layer acts as a protective barrier that occludes the dentinal tubules and prevents direct exposure of the tubule contents to intracanal irrigants [8]. Removal of the smear layer is, therefore, recommended to improve root canal disinfection [8, 9].

Most investigations on the reasons of failed endodontic treatment showed that the complex and dynamic microbial environment in the root canal system is the most common cause for failed root treated teeth with persistent periradicular disease [5, 6]. Therefore, selection of an effective antibacterial agent to use during treatment is critical. Antimicrobial solutions must possess many properties such as the ability to penetrate the infected site, to suppress or destroy microbial growth, and to avoid the possible development of resistance to the agent [10]. Hence, there is always a real need for a more potent and less harmful irrigant. Several solutions have been introduced as endodontic irrigants in attempts to effectively clean and disinfect the root canal system. Sodium hypochlorite (NaOCl), at concentrations between 0.5% and 6%, is considered to be the irrigant of choice in most endodontic treatments, as it has antimicrobial activity and the ability to dissolve both necrotic and vital tissues [11]. But according to several studies high concentration NaOCl is needed to eliminate microorganisms from the root canal [12, 13]. However, there is a concern about high concentration NaOCl for its possible toxic effect on the periapical tissues at higher concentrations [13].

**Figure 1:**
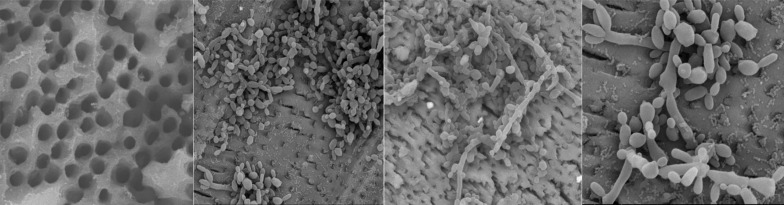
*A)* Scanning electron microscope images of roots prepared for infection with *Candida albicans*. Freshly extracted human teeth with single canals were decoronated and root apices were removed to produce 9 mm dentin cylinders. Root canals were prepared instrumentally, and smear layers removed by ultrasonic cleaning, to expose the dentinal tubules (Original magnification 5000×, HV 30.0 kV); *B)* Scanning electron microscope of *C. albicans* colonizing root dentin disc (Original magnification 4000×, HV 30.0 kV); *C)* Scanning electron microscope of *C. albicans* colonising root dentin disc (Original magnification 20000×, HV 30.0 kV); *D)* Scanning electron microscope of *C. albicans* colonising root dentin disc (Original magnification 80000×, HV 30.0 kV)

Chlorhexidine (CHX) is a potent antiseptic that possesses substantivity [14]. As a root canal irrigant, 0.12–2.0% CHX has been shown to significantly reduce the presence of micro-organisms in the root canal system [8, 15]. A mixture of doxycycline, citric acid and a detergent (MTAD) has been proposed to have antibacterial properties [16], to be biocompatible [17] and to safely remove the smear layer [18].

Propolis is a biocompatible, resinous substance that is extracted by honeybees from various plants [19]. It contains polar compounds such as flavonoids, which have antioxidant, antimicrobial and anti-inflammatory properties. The composition of propolis is complex and depends on the local flora at the site of collection [19]. A number of studies have been conducted, to explore the potential endodontic use of propolis in endodontic therapy and as a storage media with promising results [20-22]. As a root canal disinfectant, propolis has shown favorable outcomes against *Enterococcus faecalis* [23, 24] and *C. albicans* [15, 25].

To further explore the potential of propolis as an endodontic irrigant, its effectiveness against *C. albicans *in the presence or absence of the smear layer on root canal walls has now been investigated in comparison with MTAD, CHX and NaOCl. The null hypothesis tested was that no difference exists between the experimental irrigants in the eradication of *C. albicans* from the root canals in presence or absence of the smear layer.

## Materials and Methods

***Specimen preparation***

Freshly extracted human teeth with single canals (*n*=104) were collected and immediately stored in thymol solution (0.1% w/v). The teeth were decoronated using a diamond disc (Edenta AG, Au, Switzerland) to provide a standardized reference plane, and the root apices were removed to a level that produced 9 mm dentin cylinders. The root canals were prepared with the ProTaper Universal rotary system up to F3 (Dentsply Sirona, Ballaigues, Switzerland).

To remove the smear layer, the roots were immersed in a 17% aqueous solution of EDTA and subjected to ultrasonic cleaning for 10 min, followed by washing in distilled water, and 10 min ultrasonic cleaning in 2.5% NaOCl. Finally, the roots were washed thoroughly with distilled water.

***Cultivation and inoculation of C. albicans into dentin specimens***

A Culti-Loop suspension of *C. albicans *(ATCC 10231; Oxoid, Basingstoke, UK) was cultured for 48 h in 1 mL sterile Sabouraud dextrose broth (Oxoid, CM 41; Sigma Aldrich, USA) at 37^°^ C, then adjusted to a turbidity of 0.5 on the McFarland scale (corresponding to 1.5×10^8 ^cells/mL). This culture was used throughout the experiments.

***Extraction of propolis***

Propolis was collected from Northern Jordanian Valley honey farms. Propolis was extracted according to the technique described by Ansorge *et al.* [26], where 50 gr of the raw propolis were extracted by intensive stirring in a mixture of 150 mL distilled water and 250 mL chloroform at room temperature for 3 h. Phases were allowed to separate for 30 min and the aqueous phase was carefully collected and cleared by filtration through paper filters. Then, the extracts were freeze dried by lyophilization.

The resulting powder was dissolved in Dulbecco’s Modified Eagles Medium (DMEM; PAA, Austria) to yield a 300 mg/mL propolis solution. Then the solution was sterile filtered using syringe filters (Nalgene, USA). 

***Agar-diffusion test***

Sterile Mueller-Hinton agar (Oxoid, Thermofisher, Basingstoke, UK) was prepared in disposable Petri dishes 24 h before seeding with *C. albicans*. Wells of 5 mm depth and 6 mm diameter were created at the center of each agar plate and filled with 30% propolis (Nature Home, Amman, Jordan), MTAD (Dentsply Tulsa Dental Specialties, Tulsa, OK, USA), 2% CHX (Consepsis; Ultradent Products, South Jordan, UT, USA) or 3% NaOCl (ChlorCid; Ultradent Products, South Jordan, UT, USA). Plates were kept at room temperature for 10 min then incubated at 37^°^ C for 48 h. The antifungal effect of each treatment was determined by measuring the diameter of the growth-inhibition zone.

***Disinfection of infected root canals***

The 104 prepared roots were sterilized in an autoclave and then randomly allocated to four experimental groups of 20 roots each, a positive control group of 14 roots and a negative control group of 10 roots. In each group, half of the roots had a smear layer recreated by a slight instrumentation against the canal walls.

All the roots except for those in the negative control group were placed in test tubes that contained broth inoculated with *C. albicans*, and incubated at 37^°^ C for 21 days. The inoculated broth was renewed every 2-3 days. The roots in the negative control group were kept in sterile broth throughout the experiment to assess the sterilization procedure and aseptic technique.

After 21 days of incubation, each root was rinsed with a sterile saline solution and blotted dry. The external surfaces of the roots were coated with two layers of fluoride-free nail varnish (MaxFactor, Proctor & Gamble, Weybridge, UK) to avoid external contamination.

The roots were mounted in individual 22-mm diameter tissue wells (Greiner Bio-One, Cellstar®, Maybachstr, Germany) on bases of sterile melted wax. In the experimental groups, the roots were filled with the irrigant (30% propolis, MTAD, 2% CHX or 3% NaOCl), and in the positive control group they were filled with saline solution. After 5 min, the roots were washed with distilled water and a sample of dentin was taken from each root with a sterile Hedstrom file (Dentsply Maillefer, Ballaigues, Switzerland). Each sample was transferred to a sterile Eppendorf tube containing 100 μL of fresh sterile broth. Each sample was streaked onto an agar plate, and the plates were incubated aerobically at 37^°^ C for 24 h to observe any microbial growth. The colonies on the agar plates were counted with a colony counter, represented in colony-forming units (CFU) per mL.

***Preparation for SEM***

Two roots (one represents the negative control and the other one represents the positive control) were prepared as described earlier and sent for examination under scanning electron microscope (SEM; FEI Quanta 200, the Netherlands) to conﬁrm the sterility of our technique as shown in the negative control and the successful infection of the root canal as shown in the positive control.

***Statistical analysis***

Since the colony counts in the root-disinfection experiment did not follow normal distributions, the Kruskal-Wallis and Mann-Whitney tests were used to compare the overall effectiveness of different treatments at 5% significance using SPSS 17.0 (SPSS, Chicago, IL, USA).

## Results

The SEM image for sterile dentin disc confirms lack of any contamination with clear patent dentinal tubules as shown in Figure 1A. Figures 1B to D confirm *C. albicans* growth on colonization and penetration into the dentinal tubules. 

***Agar*** **-** ***diffusion test***

The inhibition zones of 30% propolis, MTAD, 2% CHX and 3% NaOCl were 20 mm, 17 mm, 50 mm and 81 mm, respectively. Although the solutions were stored according to the manufacturers’ instructions, the efficacy of NaOCl from a container that had been opened several days prior to the diffusion test differed from that of NaOCl obtained from an immediately opened container, with inhibition zones of 58 mm and 81 mm, respectively. A similar effect was seen with CHX, with inhibition zones of 33 mm for a previously opened solution and 50 mm for a freshly opened solution. This effect was not seen with propolis. 

***Disinfection of the infected root canals***

The average rank of each irrigant was calculated from the colony counts following disinfection of infected root canals (Table 1). One sample was excluded from the propolis group because colonies of different microbial species were observed on streaked agar plates, indicating a procedural contamination.

For disinfection of *C. albicans* from the roots (irrespective of the presence or absence of a smear layer), propolis, NaOCl and CHX were equally effective and all were significantly more effective than saline or MTAD solution (*P*<0.001). Propolis, NaOCl and CHX produced cultures that were free of *C. albicans *in 74%, 90% and 95% of the samples, respectively. MTAD, in common with saline solution, did not produce negative cultures in any sample.

The effectiveness of each irrigant was compared in roots with and without smear layers by comparison of the means of colony counts (Table 2). For propolis, CHX and NaOCl, no differences were seen in the colony counts of *C. albicans *in samples taken from roots in the presence or absence of the smear layer. However, for MTAD, the mean colony count was significantly lower in samples taken from roots without a smear layer than in samples from roots with a smear layer (*P*=0.04).

## Discussion

In this study, we assessed the effectiveness of endodontic irrigants against *C. albicans* in the presence or absence of the smear layer on root canal walls. Although *in vivo *testing is the most definitive method for establishing the efficacy of endodontic irrigants, *in vitro* testing has important role in the initial assessment of the antimicrobial activity of novel treatments. *In vitro* approaches to testing the antimicrobial activity of a substance include incubation with broth cultures of bacteria [27], agar-diffusion tests [28] and the disinfection of intentionally infected root canals [8].

Based on a preliminary investigation, the results of the agar-diffusion tests showed that 2% CHX and 3% NaOCl solutions from containers opened several days previously produced smaller inhibition zones than solutions from newly opened containers. This difference presumably reflects the effect of the environment on these materials after they are first used, and suggests the need for single-use doses or revised storage conditions to prevent degradation. After this preliminary investigation, only solutions from recently opened containers were used in these experiments. 

In the agar-diffusion test, propolis, MTAD, 2% CHX and 3% NaOCl produced inhibition zones of 20, 17, 50 and 81 mm, respectively. These reagents are all liquids, although propolis has a high viscosity, which could limit its diffusion. Regardless of its viscosity, propolis demonstrated effective disinfection of the infected roots (Table 1). Propolis has previously been reported to be effective against micro-organisms collected from infected roots [29]. By the criteria of minimum inhibitory concentration and minimum bactericidal concentration, propolis, MTAD, CHX and NaOCl have been shown to be effective against *C. albicans *[15]. In a study measuring zones of inhibition and minimum inhibitory concentrations, MTAD was as effective as 5.25% NaOCl against *Enterococcus faecalis* [16]. However, our results demonstrate a weak antifungal effect of MTAD against *C. albicans* compared with 3% NaOCl.

For the disinfection test, hollow dentin discs were taken from the coronal segment of the root [30], and were incubated for 21 days to enable C. albicans to colonize and penetrate the dentinal tubules [31]. The presence of broth turbidity in the positive control and experimental groups confirmed a canal infection by C. *albicans*. The sterility of the experimental procedures was confirmed by the negative control group, which showed no turbidity in the Sabouraud dextrose broth and further confirmed by SEM imaging for the negative control group (Figure 1A). 

**Table 1:** Canal disinfection with different irrigants of root canals infected with *C. albicans*

**Irrigant**	**Colony counts (CFU/mL)**				
	**Mean (SD)**	**Minimum**	**Maximum**	**Cultures free of ** ***C. albicans***	**Mean rank**
**30% Propolis (** ***n*** **=19)**	8.00 (29.00)	0	160	73.7%	34.45^b^
**MTAD (** ***n*** **=20)**	2562.00 (2586.00)	20	9000	0%	81.95^a^
**2% Chlorhexidine (** ***n*** **=20)**	1.00 (4.47)	0	20	95%	27.40^b^
**3% NaOCl (** ***n*** **=20)**	2.00 (9.00)	0	40	90%	29.15^b^
**Saline (** ***n*** **=14)**	>2300.00	300	12480	0%	81.42^a^

**Table 2:** Effect of the smear layer on the antifungal efficacy of different irrigants

**Irrigant (N)**	**With smear layer**	**Without smear layer**
	**Colony count [CFU/mL; mean (SD)]**	**Colony count [CFU/mL; mean (SD)]**
**30% Propolis (10)**	20.00 (52.92)	14.00 (25.03)
**MTAD (10)**	3606.70 (2520.8)^a^	1818.00 (2518.8)^b^
**2% Chlorhexidine (10)**	0.00 (0.00)	2.00 (6.32)
**3% NaOCl (10)**	4.00 (12.65)	4.00 (12.65)

*Different superscript letters indicated statistical significance*

Propolis, CHX and NaOCl were all effective against *C. albicans* infection of the root canal, as demonstrated by low concentrations of CFUs, and high proportions of samples free from *C. albicans* after 5 min of treatment (Table 1). By contrast, the numbers of CFUs in samples following treatment with MTAD were nearly as high as in the untreated samples of the positive controls, and none of these samples were free from *C. albicans*.

Propolis has antimicrobial, anti-inflammatory, healing, anesthetic and cariostatic properties, prevents fungal cell division and breaks down fungal cell walls and cytoplasm [32]. In our experiment, propolis treatment was completely effective in 73.7% of the samples. Previous results have also indicated that propolis is effective against *C. albicans, *and that its effectiveness is comparable with that of 2% CHX [22, 25, 33]. By contrast, weak antifungal activity has been demonstrated with Turkish propolis [34], highlighting the effect that the geographical source can have on the constituents and efficacy of this complex substance. In our root canal disinfection experiment, 2% CHX totally eliminated *C. albicans* from 95% of the samples. This finding supports the results of previous studies, which showed that 2% CHX is very effective against *C. albicans *[8, 35, 36].

In addition, a lower concentration of CHX with a long exposure (1.2% for 60 min) is sufficient to eliminate *C. albicans* from dentinal surfaces [8]. NaOCl is considered the irrigant of choice in most endodontic treatments. Our findings show that a 3% solution of NaOCl can completely disinfect 90% of root canals infected with *C. albicans* in an *in vitro* experimental system. In previous studies, NaOCl was effective to obtain an almost debris-free canal [25, 35] even at concentrations as low as 1.3% [37].

No significant differences were observed in our experiments between disinfection of root canals with and without the smear layer by propolis, CHX or NaOCl solutions. Previous studies reported that smear layer reduced the efficacy of antimicrobial agents [8, 9]. The results of a previous study suggest that the smear layer can affect the rate of disinfection of *C. albicans* by NaOCl, although in those experiments neither 0.12% CHX nor 5% NaOCl showed disinfectant activity with <1 h treatment in the presence of the smear layer [8]. Compared with these previous results [8], the much higher levels of disinfectant activity observed in our experiments with 2% CHX and 3% NaOCl (and with 30% propolis) after 5 min treatment might mask any minor effects attributable to the smear layer, which might delay but not block the action of the solutions [32].

Treatment with MTAD produced very little disinfectant activity in our system compared with the other irrigants. Evidence regarding the antifungal activity of MTAD is mixed, and although such activity has been demonstrated [15], the weak activity that we observed is consistent with other results [35, 37-39]. Moreover, Mohammadi and Asgary [39] reported that NaOCl with different concentrations and 2% CHX had better antifungal than MTAD. According to the results of previous studies [13, 40] and as recommended by the manufacturer, the comprehensive antimicrobial activity of MTAD should be enhanced by the initial use of NaOCl. In our experiments with MTAD treatment, a higher level of disinfection was seen in the absence of the smear layer than in its presence.

This study could act to determine the merit of propolis as a potential endodontic irrigant being as effective as NaOCl without the side effects of NaOCl and without the need to remove smear layer; so it could fill the unmet needs with the current recommended treatment(s); antimicrobial efficiency and organic tissue dissolving.

## Conclusion

Our findings show that propolis is a promising endodontic irrigant with comparable antimicrobial efficacy to NaOCl and CHX against *C. albicans,* even in the presence of the smear layer. Propolis effect on such a resistant micro-organism suggests that it could be beneficial in root canal treatments. MTAD was less effective in the presence of the smear layer than in its absence.
